# Supplementary material for: Feasibility assessment of double-blind, crossover, randomized controlled trial protocol comparing two oxygen-supplemented pulmonary rehabilitation for patients with chronic obstructive pulmonary disease: A pilot study
Source: PLoS One. 2026 May 7;21(5):e0348404. doi: 10.1371/journal.pone.0348404 (PMC13152130; doi:10.1371/journal.pone.0348404)
Supplement: S1 File — (PDF) [file pone.0348404.s001.pdf]

Jan 12, 2026

Feasibility assessment of double-blind, crossover, random controlled trial protocol comparing two oxygen-supplemented pulmonary rehabilitation for patients with chronic obstructive pulmonary disease

DOI
dx.doi.org/10.17504/protocols.io.x54v9bdxml3e/v1

Akihiro Ito[1], Akane Morito[2], Masahiro Ishizaka[1], Yukihiro Ogawa[2], Yuki Kawai[2], Yuta Hanawa[2], Naotaka Onodera[2], Yoshiaki Endo[1], Isato Fukushi[3], Kotaro Takeda[4], Taichi Mochizuki[5], Yasushi Inoue[5], Yasuo To[6], Kazuyuki Chibana[7], Hideaki Yamasawa[8], Satoshi Fuke[9], Sarah Kesler[10], David Gozal[11], Yasumasa Okada[12], Akira Umeda[13]

[1]Department of Physical Therapy, School of Health Science, International University of Health and Welfare;
[2]Department of Rehabilitation, International University of Health and Welfare Shioya Hospital;
[3]Graduate School of Health Sciences, Aomori University of Health and Welfare;
[4]Faculty of Rehabilitation, School of Health Sciences, Fujita Health University;
[5]Department of Respiratory Diseases Center, International University of Health and Welfare Mita Hospital;
[6]Department of General Medicine, St. Marianna University School of Medicine;
[7]Department of Pulmonary Medicine, Dokkyo Medical University;
[8]Department of Pulmonary Medicine, International University of Health and Welfare Hospital;
[9]Department of Respiratory Diseases Center, KKR Sapporo Medical Center;
[10]Intensive Care Unit, University of Minnesota;
[11]Department of Child Health and Child Health Research Institute, School of Medicine, University of Missouri;
[12]Department of Internal Medicine, Murayama Medical Center;
[13]Department of General Medicine, School of Medicine, IUHW Shioya Hospital, International University of Health and Welfare (IUHW)

Aki

Akihiro Ito

OPEN ACCESS

DOI: https://dx.doi.org/10.17504/protocols.io.x54v9bdxml3e/v1

Protocol Citation: Akihiro Ito, Akane Morito, Masahiro Ishizaka, Yukihiro Ogawa, Yuki Kawai, Yuta Hanawa, Naotaka Onodera, Yoshiaki Endo, Isato Fukushi, Kotaro Takeda, Taichi Mochizuki, Yasushi Inoue, Yasuo To, Kazuyuki Chibana, Hideaki Yamasawa, Satoshi Fuke, Sarah Kesler, David Gozal, Yasumasa Okada, Akira Umeda 2026. Feasibility assessment of double-blind, crossover, random controlled trial protocol comparing two oxygen-supplemented pulmonary rehabilitation for patients with chronic obstructive pulmonary disease. **protocols.io** **https://dx.doi.org/10.17504/protocols.io.x54v9bdxml3e/v1**

License: This is an open access protocol distributed under the terms of the **Creative Commons Attribution License**, which permits unrestricted use, distribution, and reproduction in any medium, provided the original author and source are credited

Protocol status: In development
We are still developing and optimizing this protocol

Created: January 10, 2026

Last Modified: January 12, 2026

Protocol Integer ID: 238370

Keywords: supplemented pulmonary rehabilitation, pulmonary rehabilitation for patient, pulmonary rehabilitation, oxygen supplementations during pr, oxygen supplementation during pr facilitate, copd assessment test score, patients with chronic obstructive pulmonary disease, patients with copd, chronic obstructive pulmonary disease, oxygen supplementation method, oxygen supplementation, copd, flow nasal cannula, pr for patient, physiological benefit

Funders Acknowledgements:
Akihiro Ito
Grant ID: 24K20446

Abstract

Background: Pulmonary rehabilitation (PR) for patients with chronic obstructive pulmonary disease (COPD) improves exercise tolerance and COPD assessment test score (CAT). Oxygen supplementation during PR facilitates exercise physiological benefits. This study aimed to assess the feasibility of a trial comparing two oxygen supplementation methods, with the hypothesis that both would be valid and produce distinct outcomes.
Methods: This double-blind, crossover, randomized controlled trial compared two oxygen supplementations (FiO2 0.3 or 0.5) during PR for patients with COPD (n=6) using a high-flow nasal cannula. Data on the 6MWD, CAT, and physical measurements were collected. Participants underwent one month of regular PR followed by two months of oxygen-supplemented PR, with data collected again after this period. Statistical significance was set at 0.05 with a power of 0.8, and the required sample size was calculated accordingly.
Results: The required sample size could not be calculated based on the 6MWD. The improvement in CAT by FiO2 0.3 PR was greater than that by FiO2 0.5 PR. The standardized effect size and the corresponding required sample sizes for the CAT, quadriceps muscle power, lower leg circumference, trunk muscle mass, and leg muscle mass were 0.32/81, 0.66/8, 0.17/114, 0.27/88, and 0.24/56, respectively.
Conclusions: Given the small number of participants, the 6MWD and CAT were not appropriate primary endpoints for comparing the effectiveness of the two oxygen supplementations during PR in patients with COPD. However, the quadriceps muscle power was identified as the most suitable primary endpoint among all the investigated parameters.

Image Attribution

Fig 1. Protocol. Abbreviations: 6MWD, Six-Minute Walk Distance; CAT, COPD Assessment Test; mMRC, Modified Medical Research Council Dyspnea Scale; LFT, Lung function tests; PR, pulmonary rehabilitation.

Guidelines

The treadmill speed was adjusted to target a breathing difficulty level of 4–5 on the revised Borg scale, which is a guideline for aerobic exercise, and the discontinuation criteria were based on the guidelines of the American College of Sports Medicine.

Materials

- Mobie dynamometer (SAKAI med, Japan)
- Tape measure
- Body composition analyser MC-780A-N (TANITA, Japan)
- CHESTAC-8900 respiratory function tester (CHEST, Tokyo, Japan)
- NKV-330 ventilator (Nihon Kohden)
- Transcutaneous Monitor-4 (Radiometer Medical ApS, Denmark)

Troubleshooting

Safety warnings

Concerns have been raised that both low and high flow rates could lead to issues, such as CO2 accumulation and over-reduction. Additionally, high flow rates may increase discomfort and should be considered.

Ethics statement

This double-blind, crossover, randomized controlled trial was reviewed and approved by the International University of Health and Welfare Ethics Committee according to the Declaration of Helsinki (approval number: 21-B-4). All patients provided written informed consent to participate in the study. This study is registered at the University Hospital Medical Information Network (UMIN) Clinical Trials Registry (registration number: UMIN000047507).

Introduction

1 Chronic obstructive pulmonary disease (COPD) is a common disease worldwide, with a global prevalence of more than 10%. It accounts for approximately 5% of respiratory-related deaths and is a leading cause of death in the elderly population. COPD is caused by smoking and air pollution, and is characterized by reduced alveolar ventilation due to alveolar destruction and ventilation/blood flow imbalance. This results in dyspnea, chronic cough, and increased sputum production. Additionally, it causes a decline in physical function, such as exercise tolerance and muscle weakness, and reduces quality of life.

2 Pulmonary rehabilitation (PR) plays an important role in the management of patients with COPD by improving exercise tolerance and quality of life, and reducing dyspnea and fatigue. A PR program for stable COPD is a 4- to 12-week supervised exercise program that effectively combines resistance and aerobic exercises. Oxygen therapy combined with exercise for patients with COPD has been increasingly evaluated and adopted.

3 Recently, there has been growing interest in the potential benefits of oxygen therapy with a high-flow nasal cannula (HFNC) for COPD. HFNC reduces respiratory muscle load by lowering the arterial partial pressure of carbon dioxide, increasing end-expiratory and end-tidal volumes, and decreasing the respiratory rate, which may improve respiratory patterns. Rehabilitation with HFNC has been shown to be superior to conventional rehabilitation using a nasal cannula or Venturi mask in improving the 6-minute walk distance (6MWD) and exercise endurance time in patients with COPD. Additionally, exercise training with HFNC may be superior to exercise training with a regular nasal cannula in patients with chronic respiratory failure receiving long-term oxygen therapy.

4 However, most of these studies have focused on in-situ observations with few interventional studies. Additionally, the optimal setting for HFNC has not been adequately investigated. A meta-analysis of HFNC intervention effects found that while some improvements in quality of life and exercise tolerance were observed, the evidence was insufficient. This lack of consistency was partly due to varying HFNC settings across studies.

5 Therefore, this study aimed to assess the feasibility of a trial comparing HFNC at two FiO2 levels, with the hypothesis that both methods would be valid and differ from each other. This study may maximize the effects of PR, further improve patients' exercise capacity and quality of life, and reduce dyspnea. Moreover, identifying the optimal HFNC setting could enhance its clinical utility and provide a new standard of care for patients with COPD.

Materials and methods

6 This double-blind, crossover, randomized controlled trial was reviewed and approved by the International University of Health and Welfare Ethics Committee according to the Declaration of Helsinki (approval number: 21-B-4). All patients provided written informed consent to participate in the study. This study is registered at the University Hospital Medical Information Network (UMIN) Clinical Trials Registry (registration number: UMIN000047507).

7 Patients with COPD were recruited from the International University of Health and Welfare (IUHW) Shioya Hospital. The diagnosis of COPD was based on forced spirometry showing the presence of a post-bronchodilator forced expiratory volume in 1 s (FEV1)/forced vital capacity (FVC) 3c 0.7. Six participants were consecutively recruited for the pilot study from February 2022 to August 2022. At least nine months per participant were required to record the full data.

8 Study participants were randomly assigned to one of the two groups by their physicians, with one group starting FiO2 0.3 PR and the other starting FiO2 0.5 PR (Figure 1). These assignments were unknown to the physical therapist in charge of PR.

9 The study protocol is illustrated in Figure 1. The participants underwent regular rehabilitation without oxygen supplementation once a week for four sessions (approximately 1 month). They then underwent eight PR sessions once a week (approximately 2 months) with assigned oxygen supplementation. After the completion of the intervention, a washout period of 3e 3 months was established. Following the washout period, PR was performed once a week for 12 sessions (approximately 2 months) with crossover oxygen supplementation.

10 A total of six assessments were performed: at baseline, after normal rehabilitation, following the washout period, and at the end of PR. Evaluations included 6MWD, COPD assessment test (CAT), modified Medical Research Council (mMRC) dyspnea scale, respiratory function, quadriceps strength at knee extension, lower leg circumference, and body composition.

11 The 6MWD was measured once, following the protocol from previous studies [28, 29]; however, the distance for a walking round trip was set to 15 m in this study. Participants walked at their own pace in comfortable clothing, aiming to cover as much distance as possible in 6 min. Rest breaks were allowed during walking, and the physiotherapist in charge encouraged participants to rest if subjective fatigue, dyspnea, or a significant drop in SpO2 (below 90%) were observed. Participants were instructed to walk slowly if SpO2 fell below 85%, and to stop if necessary. Blood pressure, respiratory rate, heart rate, dyspnea, and lower limb fatigue were measured before and after the measurements using the Borg scale.

12 CAT and mMRC scores were obtained directly from participants using specialized measurement forms [30, 31]. Lower scores indicate better conditions for both scores.

13 The knee extensor strength was measured using a Mobie dynamometer (SAKAI med, Japan). The participant was seated with the knee joint flexed at 90°, and a band was attached to the distal leg. Isometric contraction was used to assess bilateral muscle strength [32]. Measurements were taken twice, and the maximum value was used.

14 The circumference of the lower leg was measured using a tape at the point of maximum circumference. Measurements were taken three times, and the median value was used [33].

15 Body composition was measured using a body composition analyser MC-780A-N (TANITA, Japan). The participants were assessed in a standing position with their feet bare. The measurement periods were set to be the same. Limb and trunk site-specific total muscle mass, body fat percentage, body fat mass, and lean body mass were measured.

16 Respiratory function was measured using a CHESTAC-8900 respiratory function tester (CHEST, Tokyo, Japan). Lung capacity and forced vital capacity tests were performed using standardized methods [34, 35]. The reference values for the FEV1 predicted values were derived from the Japanese standards [36].

17 The range of motion (trunk flexion and extension) was measured using the methods of the Japanese Orthopaedic Association, Japanese Association of Rehabilitation Medicine, and Japanese Society of Foot Surgery. Measurements were taken from the lateral aspect of the trunk, with the posterior sacrum as the base axis and the line connecting the first thoracic spinous process and the fifth lumbar spinous process as the axis of motion. Care was taken to ensure that hip motion was not included.

Pulmonary rehabilitation under O2 supplementation with HFNC

18 The PR protocol consisted of conditioning, resistance exercise (approximately 20 min), resting on a chair, and walking on a treadmill for 20 min under O2 supplementation with HFNC. PR was conducted for approximately 40 min once a week. Conditioning focused on the relaxation and stretching of the thoracic and respiratory muscles. Resistance exercises were performed mainly on the lower extremities while monitoring for dyspnea. The treadmill speed was adjusted to target a breathing difficulty level of 4–5 on the revised Borg scale, which is a guideline for aerobic exercise, and the discontinuation criteria were based on the guidelines of the American College of Sports Medicine [37]. Oxygen supplementation was provided using a NKV-330 ventilator (Nihon Kohden), with a flow rate set at 20 L/m unless otherwise indicated. The FiO2 was set at 0.3 or 0.5. HFNC was initiated just prior to the start of conditioning and discontinued after the end of the walking exercise on a treadmill, when the participant was resting in a sitting position.

19 During the rehabilitation intervention, the Transcutaneous Monitor-4 (Radiometer Medical ApS, Denmark) was used to monitor PtcCO2 and PtcO2. Since PtcCO2 values

are typically higher than $PaCO_2$ values, many commercially available instruments have adopted a post-measurement adjustment approach, such as subtracting 4–5 mmHg from the directly measured $PtcCO_2$ values for better estimation of the adult $PaCO_2$. However, we did not adopt such an adjustment; both $PtcCO_2$ and $PtcO_2$ were recorded directly from the tcSensor 84.

20 This approach was applied consistently across all subject runs [38, 39, 40]. Skin sensors were applied to the forearms of the participants following the manufacturer's recommendations.

Safety of HFNC settings

21 Previous studies on respiratory rehabilitation interventions using HFNC have employed flow rates ranging from 20 to 60 L/min, which is inconsistent [26]. Concerns have been raised that both low and high flow rates could lead to issues, such as CO_2 accumulation and over-reduction. Additionally, high flow rates may increase discomfort and should be considered [41]. Based on the results of previous studies, $PtcCO_2$ and $PtcO_2$ were monitored during rehabilitation using the Transcutaneous Monitor, with flow rates between 20 and 40 L/min. At lower flow rates (20 L/min), no issues with elevated $PtcCO_2$ were observed. However, at higher flow rates (40 L/min), $PtcCO_2$ dropped significantly during and after exercise therapy, and discomfort was reported (Figure 2). Therefore, the flow rate in this study was set at 20 L/min.

22 (A) O_2 loading(Flow rate 40L/m) Stretching and Resistance training begins. (B) Finish stretching and resistance training. (C) Aerobic exercise begins. (D) Finished aerobic exercise. (a) It was low during aerobic exercise. Seventeen minutes and 30 seconds after the start of the treadmill, the patient complained of dizziness and it was stopped. (b) There was a decrease after the exercise was stopped. Subject complained of feeling dizzy.

Calculation of the number of subjects needed

23 To determine the required sample size, standardized effect sizes were calculated [42], targeting a significance level of 0.05 and power of 0.8. We assumed that both methods (FiO_2 0.3 and 0.5) had a direction of validity and would show differences in effectiveness. Initially, the 6MWD was selected as the primary endpoint; however, as the study progressed, we reconsidered its suitability due to cases where 6MWD showed no improvement after PR. Our aim then shifted to identifying a more appropriate indicator that demonstrated significant gains post-PR to use in calculating the required sample size. A larger standardized effect size corresponds to a smaller sample size, indicating better feasibility [42]. The change in FiO_2 0.5 PR compared to that in FiO_2 0.3 PR was defined as the effect size (E), and the standard deviation (S) was calculated from all measurements in the six patients with COPD. The standardized effect size was then calculated as E/S.

Statistical analysis

24 Data are expressed as mean ± standard deviation unless otherwise indicated. Student’s paired t-test compared between baseline and after walking rehabilitation (two-tailed). Additionally, a sub-analysis was conducted to assess correlations among evaluation indices using Spearman’s rank correlation coefficient. Statistical significance was set at p [illegible]c 0.05. Commercially available statistical software (BellCurve for Excel; Social Survey Research Information Co., Ltd., Tokyo, Japan) and SPSS version 25 (IBM Corp., Armonk, NY, USA) were used for the statistical analyses.

Protocol references

[1] Global prevalence of COPD. [2] Alveolar destruction and ventilation/blood flow imbalance. [3] Increased sputum production. [4, 5] Decline in physical function. [6-12] PR program benefits. [13] Supervised exercise program. [14, 15] Resistance and aerobic exercises. [16-19] Oxygen therapy evaluation. [20-22] HFNC benefits. [23, 24] 6MWD improvement. [25] Exercise training with HFNC. [26] Meta-analysis of HFNC intervention effects. [27] Forced spirometry diagnosis criteria. [28, 29] Protocol for 6MWD measurement. [30, 31] Measurement forms for CAT and mMRC scores. [32] Mobie dynamometer use. [33] Lower leg circumference measurement. [34, 35] Lung capacity and forced vital capacity tests. [36] Japanese standards for FEV1 predicted values. [37] Guidelines of the American College of Sports Medicine. [38, 39, 40] Consistent application of Transcutaneous Monitor-4. [41] Considerations for high flow rates. [42] Sample size calculation.
