# Supplementary material for: Feasibility assessment of double-blind, crossover, randomized controlled trial protocol comparing two oxygen-supplemented pulmonary rehabilitation for patients with chronic obstructive pulmonary disease: A pilot study
Source: PLoS One. 2026 May 7;21(5):e0348404. doi: 10.1371/journal.pone.0348404 (PMC13152130; doi:10.1371/journal.pone.0348404)
Supplement: S2 File — Data on lung function tests are shown in the latter part of this table. *: p < 0.05, **: p < 0.01, ***: p < 0.001. Abbreviations: 6MWD, Six-Minute Walk Distance; CAT, COPD Assessment Test; mMRC, Modified Medical Research Council Dyspnea Scale; BMI, Body Mass Index; VC, Vital Capacity; ERV, Expiratory Reserve Volume; IRV, Inspiratory Reserve Volume; TV, Tidal Volume; IC, Inspiratory Capacity; FVC, Forced Vital Capacity; FEV1.0, Forced Expiratory Volume in 1 Second; PEF, Peak Expiratory Flow; V75, Expiratory Flow at 75% of Forced Vital Capacity; V50, Expiratory Flow at 50% of Forced Vital Capacity; V25, Expiratory Flow at 25% of Forced Vital Capacity; MMF, Maximal Mid-Expiratory Flow. (DOCX) [file pone.0348404.s002.docx]

Supplementary Material

**S2 Table. Changes before and after oxygen-supplemented pulmonary rehabilitation.**

| Item | Before | After | P-value |  |
| --- | --- | --- | --- | --- |
| 6MWD (m) | 278.3 ± 96.6 | 290.9 ±122.6 | 0.53 |  |
| MinSpO2 during 6MWT (%) | 82.3 ± 7.2 | 82.4 ± 6.0 | 0.92 |  |
| 6MWTBorg (Lower limb) | 2.2 ± 2.0 | 1.0 ± 1.1 | 0.073 |  |
| 6MWTBorg (Respiratory) | 3.1 ± 1.7 | 2.5 ± 0.2 | 0.15 |  |
| CAT | 16.9 ± 7.9 | 10.0 ± 5.9 | 0.011 | * |
| mMRC | 2.3 ± 0.9 | 2.3 ± 0.8 | 1 |  |
| Muscle power (Truncus) | 4.7 ± 0.7 | 5.0 ± 0.0 | 0.1 |  |
| Muscle power (Rt Quad) | 25.0 ± 8.4 | 29.9 ± 10.0 | 0.026 | * |
| Muscle power (Lt Quad) | 25.1 ± 10.3 | 29.0 ± 11.2 | 0.023 | * |
| Muscle power (Rt or Lt Quad) | 25.1 ± 9.2 | 29.4 ±10.4 | 0.0011 | ** |
| Rt Lower leg circumference (cm) | 32.5 ± 2.5 | 33.0 ± 2.8 | 0.0057 | ** |
| Lt Lower leg circumference (cm) | 32.5 ± 2.7 | 33.1 ± 2.8 | 0.0019 | ** |
| Rt or Lt Lower leg circumference (cm) | 32.5 ± 2.6 | 33.1 ± 2.8 | 0.000018 | *** |
| Range of motion (truncus flex) | 43.8 ± 8.0 | 48.3 ± 7.8 | 0.085 |  |
| Range of motion (truncus extention) | 27.1 ± 9.6 | 26.7 ± 7.2 | 0.86 |  |
| Range of motion (Rt truncus parietal flexion) | 27.9 ± 4.0 | 28.8 ± 4.8 | 0.5 |  |
| Range of motion (Lt truncus parietal flexion) | 32.5 ± 9.4 | 30.4 ± 7.2 | 0.32 |  |
| Range of motion (Rt trunk rotation | 49.2 ± 6.7 | 48.3 ± 9.1 | 0.67 |  |
| Range of motion (Lt trunk rotation) | 49.6 ± 7.5 | 52.1 ± 11.6 | 0.31 |  |
| Weght (kg) | 54.6 ± 6.6 | 54.9 ± 7.2 | 0.33 |  |
| Height (cm) | 158.9 ± 6.5 | 158.9 ± 6.5 | 0.34 |  |
| BMI | 22.0 ± 2.0 | 21.9 ± 2.2 | 0.78 |  |
| Body fat percentage (%) | 31.6 ± 7.1 | 30.6 ± 8.1 | 0.27 |  |
| Fatty constitution (kg) | 17.3 ± 4.9 | 17.1 ± 5.8 | 0.6 |  |
| Lean body mass (kg) | 37.3 ± 5.4 | 37.8 ± 5.3 | 0.082 |  |
| Muscle mass (kg) | 35.3 ± 5.1 | 35.8 ± 5.0 | 0.074 |  |
| Muscle mass (trunk) (kg) | 20.2 ± 2.6 | 20.4 ± 2.5 | 0.026 | * |
| Muscle mass, right arm (kg) | 1.80 ± 0.42 | 1.79 ± 0.04 | 0.87 |  |
| Muscle mass, lt arm (kg) | 1.69 ± 0.33 | 1.70 ± 0.33 | 0.85 |  |
| Muscle mass, rt or lt arm (kg) | 1.75 ± 0.37 | 1.75 ± 0.36 | 1 |  |
| Muscle mass, right leg (kg) | 5.76 ±1.22 | 5.94 ± 1.22 | 0.026 | * |
| Muscle mass, lt leg (kg) | 5.78 ± 1.19 | 5.93 ± 1.17 | 0.053 |  |
| Muscle mass, rt or lt leg (kg) | 5.77 ± 0.34 | 5.94 ± 0.34 | 0.0024 | ** |
| Lung function tests | Before | After | P-value |  |
| VC(L) | 2.84 ± 0.59 | 2.89 ± 0.70 | 0.5 |  |
| %VC(%) | 105.0 ± 16.7 | 106.1 ± 18.0 | 0.62 |  |
| ERV(L) | 0.86 ± 0.40 | 0.88 ± 0.39 | 0.86 |  |
| %ERV(%) | 77.5 ± 30.1 | 78.9 ± 31.7 | 0.86 |  |
| IRV(L) | 1.01 ± 0.29 | 1.15 ± 0.31 | 0.15 |  |
| TV | 0.97 ± 0.35 | 0.86 ± 0.41 | 0.24 |  |
| IC(L) | 1.98 ± 0.40 | 2.00 ± 0.49 | 0.8 |  |
| FVC(L) | 2.59 ± 0.64 | 2.58 ± 0.74 | 0.88 |  |
| %FVC(%) | 95.4 ± 19.4 | 94.3 ± 19.9 | 0.67 |  |
| FEV1.0(L) | 1.45 ± 0.56 | 1.43 ± 0.58 | 0.42 |  |
| %FEV1(%) | 79.5 ± 30.5 | 68.5 ± 36.7 | 0.27 |  |
| FEV1/FVC(%) | 54.9 ± 11.1 | 54.2 ± 10.9 | 0.53 |  |
| PEF(L/S) | 4.14 ± 1.64 | 4.11 ± 1.67 | 0.85 |  |
| %PEF(%) | 53.1 ± 19.8 | 52.7 ± 19.6 | 0.81 |  |
| V75(L/s) | 2.18 ±1.25 | 2.00 ± 1.24 | 0.16 |  |
| %V75(%) | 36.2 ± 19.3 | 33.6 ± 20.5 | 0.22 |  |
| V50(L/s) | 0.77 ± 0.40 | 0.77 ± 0.44 | 0.92 |  |
| %V50(%) | 19.8 ± 9.6 | 19.8 ± 11.0 | 1 |  |
| V25(L/s) | 0.32 ± 0.14 | 0.28 ± 0.11 | 0.15 |  |
| %V25(%) | 29.4 ± 13.4 | 25.4 ± 9.6 | 0.18 |  |
| V25/HT | 0.20 ± 0.08 | 0.17 ± 0.07 | 0.12 |  |
| %V25/HT | 21.5 ± 8.2 | 18.8 ± 6.9 | 0.16 |  |
| V50/V25 | 2.46 ± 0.59 | 2.93 ± 0.86 | 0.88 |  |
| MMF(L/s) | 0.67 ± 0.34 | 0.62 ± 0.31 | 0.24 |  |
| %MMF | 28.0 ± 12.5 | 26.3 ± 12.4 | 0.34 |  |

Data on lung function tests are shown in the later part of this table. *: p < 0.05, **: p < 0.01, ***: p < 0.001.
Abbreviations: 6MWD, Six-Minute Walk Distance; CAT, COPD Assessment Test; mMRC, Modified Medical Research Council Dyspnea Scale; BMI, Body Mass Index; VC, Vital Capacity; ERV, Expiratory Reserve Volume; IRV, Inspiratory Reserve Volume; TV, Tidal Volume; IC, Inspiratory Capacity; FVC, Forced Vital Capacity; FEV1.0, Forced Expiratory Volume in 1 Second; PEF, Peak Expiratory Flow; V75, Expiratory Flow at 75% of Forced Vital Capacity; V50, Expiratory Flow at 50% of Forced Vital Capacity; V25, Expiratory Flow at 25% of Forced Vital Capacity; MMF, Maximal Mid-Expiratory Flow.
