# Supplementary material for: Feasibility assessment of double-blind, crossover, randomized controlled trial protocol comparing two oxygen-supplemented pulmonary rehabilitation for patients with chronic obstructive pulmonary disease: A pilot study
Source: PLoS One. 2026 May 7;21(5):e0348404. doi: 10.1371/journal.pone.0348404 (PMC13152130; doi:10.1371/journal.pone.0348404)
Supplement: S4 File — Abbreviations: 6MWD, Six-Minute Walk Distance; CAT, COPD Assessment Test; mMRC, Modified Medical Research Council Dyspnea Scale; Rt, Right; Lt, Left; BMI, Body Mass Index. (DOCX) [file pone.0348404.s004.docx]

Supplementary Material

**S4 Table. Spearman's rank correlation coefficient results between each pre-post change measure.**

**
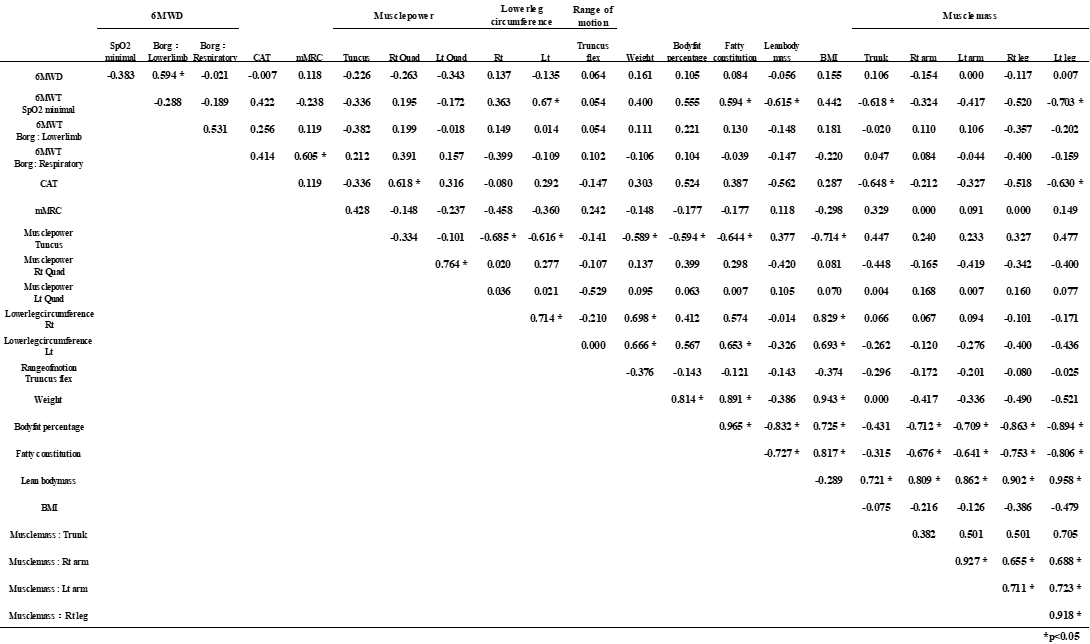
**

Abbreviations: 6MWD, Six-Minute Walk Distance; CAT, COPD Assessment Test; mMRC, Modified Medical Research Council Dyspnea Scale; Rt, Right; Lt, Left; BMI, Body Mass Index.
